# Far Out! Foot-and-Mouth, Ribosomes, and Other Tales

**DOI:** 10.1100/tsw.2001.42

**Published:** 2001-03-31

## Abstract

The spreading scourge of foot-and-mouth disease heads Nature's news this week. Science leads this week with a story about stunning new images of ribosomes.

Delays in identifying infected sheep exaggerated the recent outbreak of foot-and-mouth disease in the UK, reports *Nature* in this week's lead story. Three epidemiological studies now conclude that sheep at a Newcastle farm in the north of England were infected with the foot-and-mouth virus in early February. But the disease, which is not harmful to humans but lowers milk and meat production in infected animals, was not discovered until the animals had traveled 400 km to a slaughterhouse in Essex, reports *Nature*. Restrictions on travel were instituted as soon as the sheep were diagnosed, but not before the virulently contagious disease had infected animals destined for the far corners of England. The three studies all concluded that the foot-and-mouth epidemic is out of control, reports *Nature*, and estimated that at least 600,000 animals may be killed to eradicate the virus. The shockingly high numbers are leading some epidemiologists to suggest drastic measures — like vaccination! Farmers prefer not to vaccinate, says *Nature*, because many countries refuse to import vaccinated animals.

Although DNA has been getting all the press lately, it is the ribosomes that do the gritty work of stitching together amino acids to produce proteins from the instructions encoded in DNA. Understanding the process has proven difficult, mostly because the scientists couldn't see the action. Now, molecular biologists have produced the first molecular image of an entire ribosome, reports *Science* in their lead story. And even better, they caught the tiny factories at work. The biologists discovered that transfer RNA (tRNA), which carry amino acids through the ribosome, move as much as 50 Å while they deposit amino acids on the growing protein chain. That is a long way for a few-angstrom-long molecule to go, say the scientists interviewed by *Science*. How exactly the ribosome orchestrates this dance remains fuzzy, reports *Science*, but biologists are hopeful that future higher resolution images will reveal all.

Short-term memory loss may be a common complaint among pot smokers and lab rats dosed with cannabinoids, the active ingredient in marijuana, but the "endogenous cannabinoids" produced in our brains may be necessary for laying down memory, reports *Science*. Three independent research teams — from the University of California, San Francisco (UCSF), Kanazawa University School of Medicine in Japan, and Harvard Medical School in Boston — have shown that cannabinoids are produced to fine-tune the signals received by the hippocampus, a brain area involved in memory, and in the cerebellum, which coordinates movements. Far out, dude!

Cannabis also made news in *Nature* this week. In 1998, several clinical trials of cannabis began to test marijuana for use in treating diseases like Multiple Sclerosis. But now the UK Medicines Control Agency (MCA) is insisting on full toxicology testing of cannabis extracts in clinical trials, reports *Nature*. Researchers are accusing the MCA of slowing down testing by ignoring accumulated knowledge of the drug's safety. Far out, dude! Wait...did I already say that?
